# Proteome analysis of the *Escherichia coli *heat shock response under steady-state conditions

**DOI:** 10.1186/1477-5956-7-36

**Published:** 2009-09-21

**Authors:** Svenja Lüders, Claas Fallet, Ezequiel Franco-Lara

**Affiliations:** 1 Institute of Biochemical Engineering, Technische Universität Braunschweig, Gausstrasse 17, 38106 Braunschweig, Germany

## Abstract

In this study a proteomic approach was used to investigate the steady-state response of *Escherichia coli *to temperature up-shifts in a cascade of two continuously operated bioreactors. The first reactor served as cell source with optimal settings for microbial growth, while in the second chemostat the cells were exposed to elevated temperatures. By using this reactor configuration, which has not been reported to be used for the study of bacterial stress responses so far, it is possible to study temperature stress under well-defined, steady-state conditions. Specifically the effect on the cellular adaption to temperature stress using two-dimensional gel electrophoresis was examined and compared at the cultivation temperatures of 37°C and 47.5°C. As expected, the steady-state study with the double bioreactor configuration delivered a different protein spectrum compared to that obtained with standard batch experiments in shaking flasks and bioreactors. Setting a high cut-out spot-to-spot size ratio of 5, proteins involved in defence against oxygen stress, functional cell envelope proteins, chaperones and proteins involved in protein biosynthesis, the energy metabolism and the amino acid biosynthesis were found to be differently expressed at high cultivation temperatures. The results demonstrate the complexity of the stress response in a steady-state culture not reported elsewhere to date.

## Background

Microorganisms live in a permanently changing environment being the temperature variation one of the most important existing stress factors. For instance, a temperature increase induce the bacterial heat shock response which allows cells to adapt and survive thermal stress conditions [1-4]. Nowadays the heat shock response is of importance for many scientific and industrial applications, e.g. in processes where temperature-induced heterologous protein production takes place [5].

The general heat shock response was first discovered in *Drosophila sp*. by Rotissa, who suggested that cells exposed to heat induce the synthesis of a well-defined number of proteins [6-8]. The heat shock response in *E. coli *was discovered by the Neidhardt and Yura groups. They used one-dimensional or two-dimensional gels to detect temperature-induced proteins using batch experiments in shaking flasks [9,10]. After a temperature up-shift the *E. coli *heat shock response induced the synthesis of more than 20 heat shock proteins which protected the cell against thermal damage [1,3]. Typical heat shock proteins are chaperones and proteases which promote protein folding, refolding, quality control and protein degradation [11,12] Furthermore, it was demonstrated that most of the heat shock genes of *E. coli *are under the control of the alternative sigma factor σ^32 ^(σ^H^)[13]. Another alternative sigma factor involved in the heat shock response is σ^24 ^(σ^E^), which was found to be an essential gene in *E. coli *at all investigated temperatures [14]. In addition, many of the heat shock proteins were required during normal cell growth [10,15-17].

*E. coli *is able to grow over a range of approximately 40°C. The normal temperature growth range is located from 21°C to 37°C. The maximum temperature at which balanced growth can occur is approximately 49°C. The growth rate of several strains of *E. coli*, including K-12 strains, is markedly influenced in the high temperature range (40-45°C) by the availability of exogenous methionine [18]. In the absence of methionine, growth stops at 45°C. Between 40°C and 45°C, the growth rate is limited by the absence of methionine. At these temperatures the activity of the first enzyme of the methionine biosynthetic pathway, the homoserine transsuccinylase, is inhibited [19]. For these reasons, the control of the methionine synthesis regulates many aspects of growth, since it appears to be the most temperature-sensitive synthesis pathway in *E. coli *[18].

Even though the heat shock response has been profoundly investigated in *E. coli *(reviewed in [20]), surprisingly most of the research done so far has been reported on the basis of the heat shock response carried out in batch experiments in shaking flasks or bioreactors. The choice of batch experiments presents several disadvantages, since it leads to different transient nutrient and oxygen availability conditions during cultivation. Moreover, in shaking flask cultivations the pH is usually uncontrolled. Contrastingly, the growth conditions at steady-state are well-defined and constant with the additional advantage to adjust the growth rate, via the dilution rate control, allowing the study of stress susceptibility and growth separately. Only few reports are known using a continuous cultivation approach. Hasan and Shimizu [21] investigated the temperature up-shift effect on fermentation and metabolic characteristics considering the gene expression in *E. coli*. They used batch and continuous cultivations to study the heat shock response in a time dependent manner.

In this work we used the proteomic approach to study the steady-state response of *E. coli *to a temperature up-shift in a cascade of two continuously operated bioreactors. The first reactor served as cell source with optimal settings for *E. coli *growth at a cultivation temperature of 37°C and a constant dilution rate of 0.225 h^-1^. In the second chemostat the cells were exposed to an elevated temperature (47.5°C) decoupling the heat shock response from any other transient process parameter and therefore enabling the proteome investigation under constant and reproducible conditions.

Both culture systems are considered in this study as perfectly mixed, ideal continuous-flow stirred-tank bioreactors and their operation can be well characterized by the dilution rate *D*, defined by:

(1)

where *F *is the volumetric flow rate of feed and effluent liquid streams and *V*_*R *_the total volume of culture within the respective bioreactor. However, while in the growth bioreactor (System 1) a straightforward interpretation of the dilution rate in term of the kinetics of balanced growth can be given, i. e. the specific growth rate equals the dilution rate [22], the operation in the stress bioreactor (System 2) and the consequent heat shock response must be interpreted differently.

The dilution rate *per se *is equal to the number of tank liquid volumes which pass through the bioreactor per unit time. This is also the reciprocal of the mean holding time or mean residence time, τ:

(2)

τ can be interpreted as the expected or average time that any species (reactants, microorganisms, fluid elements, etc.) will spend in the bioreactor during continuous operation [22-24]. For this reason, any experiments in the stress reactor can be better explained in terms of the mean residence time. In the case of our stress bioreactor and considering an integral residence time distribution of the form [23]:

(3)

this would mean that more than 99% of the cells present in the vessel are exposed to a temperature of 47.5°C for a period of time equal to the mean residence time after reaching stationary conditions (*t *≥ 5 τ). Since the heat shock response is not only a function of the stress intensity but also a function of the duration of stress, our two bioreactors platform offers a possibility to investigate stress intensity and duration independently from each other varying temperature level and dilution rate (mean residence time) respectively.

In our study a dilution rate of 0.2475 h^-1 ^was used. This corresponds to a mean residence time of 4.04 h, i. e. the cells were incubated in average for more than 4 h at the higher cultivation temperature in the stress bioreactor. In contrast to previous studies it was shown that the synthesis of the heat shock proteins accelerates in the first seconds after the temperature increase, lasted no more than 20 min and reached a steady-state level [25,26]. On the basis of this fact the proteomic analysis of the *E. coli *heat shock response under steady-state conditions were analysed and any inhomogeneities can be neglected since only a marginal amount of the *E. coli *cells have lower or bigger exposure times at this heat shock condition.

## Results and Discussion

### Biochemical analysis of the steady-state cultivations

Considering an ideal well-stirred bioreactor under steady-state conditions any biochemical and physiological parameter of the performed experiments was considered to remain constant for all cultivations. Glucose was present at a limiting concentration in the stress bioreactor at the cultivation temperature of 47.5°C (Table 1). At 47.5°C no growth could be detected and the glucose was completely depleted, however, at 47.5°C high amounts of acetate (15.9 g L^-1^) were synthesized. The assimilated glucose was most probably used for the maintenance metabolism to adapt and survive the stress conditions. Growing on glucose as carbon source, *E. coli *produces and excretes acetate which may serve as well as an additional carbon source. This phenomenon is known as overflow metabolism or 'bacterial Crabtree effect' [27-33].*E*. *coli *produces acetate at glucose concentrations above about 30 mg/l [33] which inhibits the growth even under neutral pH [34]. The protonated form of acetate crosses the cell membrane [35,36] and deprotonates in the cytoplasm decreasing the intracellular pH [36,37]. In addition to acetate the other detected by-product, formate, was observed in increased concentration with increasing cultivation temperature. At 37°C, 0.43 g/l formate was measured in the growth bioreactor while at 47.5°C, 2.35 g/l was measured in the stress bioreactor.

**Table 1:** Glucose, acetate and formate concentration in the growth bioreactor at 37°C and the stress bioreactor at 47.5°C.

**Metabolite**	**Growth bioreactor** **37°C**	**Stress bioreactor** **47.5°C**
glucose	0.019 ± 0.013	0.025 ± 0.035
acetate	0.098 ± 0.098	15.870 ± 0.025
formate	0.428 ± 0.236	2.354 ± 1.200

### Proteomic analysis of the steady-state heat shock response

The proteome analysis was carried out using three different extraction methods for the protein preparation in addition to the normal cytoplasmic protein preparation to enrich hydrophobic membrane proteins and extend the protein spectrum of the steady-state heat shock response. The first method uses triton-X-114 to extract membrane proteins. Substances such as triton are molecular hybrids which are composed of a hydrophilic and a hydrophobic part. This enables the hydrophobic membrane proteins to be dissolved in water. In the second method the cell membranes were treated with sodium carbonate at pH 11.5. This treatment gives a negative charge to the proteins and the cell membrane. The charge affects directly those proteins that are not closely attached to the cell membrane and repel from this, which are also negatively charged, and can be dissolved for further analysis. The third method uses a rehydration buffer containing thiourea, urea, CHAPS, DTT, Triton and ASB-14 to dissolve membrane proteins and membrane attached proteins. All results obtained in the study are summarized in Table 2 and will be discussed in the following.

**Table 2:** Proteins identified which show a 5-fold up- or down-regulation under temperature stress at 47.5°C in the stress bioreactor compared to the reference system, the growth reactor at 37°C.

				**normalized spot intensity**	
**protein group**	**description**	**function**	**accession number**	**growth bioreactor**	**stress bioreactor**

**enzymes involved in oxidative stress**				37°C	47.5°C

Dps	DNA-binding protein	protection of DNA	NP_415333.1	1 ± 0.024	7.32 ± 0.479
AhpC	reductase subunit C	formation of H_2_O from H_2_O_2_	NP_415138.1	1 ± 0.068	17.74 ± 1.203
SodA	superoxide dismutase	convertion of O_2_- to H_2_O_2_	NP_418344.3	1 ± 0.006	17.74 ± 0.11
**proteins of the cell envelope**					

FnlC	l-fucosamine synthetase	O antigen biosynthesis	Q5ISY2	20.5 ± 4.24	1 ± 0.707
OmpA	outer membrane protein A	membrane transport	NP_415477.1	42 ± 0.004	2 ± 0.606
OmpF	outer membrane protein F	membrane transport	NP_415449.1	1 ± 0.188	31.47 ± 1.425
OmpF	outer membrane protein C+F	membrane transport	NP_415449.1	1 ± 0.078	28.47 ± 2.21
Ag43	antigen 43	autoaggregation	AP_002599.1	1 ± 0.324	7.57 ± 10.212
**chaperones and proteins involved in protein biosynthesis**					

Skp	chaperone protein	periplasmic chaperone	NP_414720.1	1 ± 0.707	33.92 ± 0.354
OppA	oligopeptide-binding protein	chaperone	NP_415759.1	8.77 ± 0.362	1 ± 0.099
Hsp40	chaperone protein	cytoplasmic chaperone	NP_414556.1	1 ± 0.098	5.45 ± 0.536
LeuRS	leucyl-tRNA synthetase	linking the amino acid to its tRNA	NP_415175.1	1 ± 0.046	4.67 ± 0.213
RP S2	ribosomal protein S2	translation	NP_414711.1	16.51 ± 3.29	1 ± 0.164
RP S4	ribosomal protein S4	translation	NP_417755.1	1 ± 0.561	7.4 ± 1.212
Rp L5	ribosomal protein L5	translation	NP_417767.1	1 ± 0.326	5.45 ± 0.427
Rp S11	ribosomal protein S11	translation	NP_417756.1	1 ± 0.09	6.53 ± 1.65
**enzymes involved in the glycolysis, TCA cycle and mixed acid fermentation**					

TpiA	triosephosphate isomerase	glycolysis	NP_418354.1	1 ± 0.062	6.4 ± 0.4
GpmA	2,3-bisphosphoglycerate-dependent phosphoglycerate mutase	glycolysis	NP_415276.1	1 ± 0.068	16 ± 1.09
GltA	citrate synthase	TCA cycle	NP_415248.1	1 ± 0.036	7.88 ± 0.28
AcnB	malate synthase	TCA/glyoxylate cycle	NP_414660.1	1 ± 0.25	0
AceB	aconitase B	glyoxylate cycle	NP_418438.1	1 ± 0.311	5.55 ± 1.73
Pta	acetyl-CoA:Pi acetyltransferase	acetate formation and dissimilation	NP_416800.1	1 ± 0.25	0
AtpG	F0F1 ATP synthase subunit gamma	ATP synthesis	NP_418189.1	1 ± 0.413	7.05 ± 0.48
**enzymes involved in amino acid biosynthesis**					

MetE	triglutamate-homoserine methyltransferase	methionine biosynthesis	NP_418273.1	1.04 ± 0.199	9.74 ± 0.282
FolE	GTP cyclohydrolase I	tetrahydrofolate biosynthesis	NP_416658.1	20.33 ± 0.471	3.33 ± 0.943
CysP	thiosulfate transporter	uptake of sulphate and thiosulfate	NP_4168920.1	1 ± 0.188	4.17 ± 2.71
MetN	DL-methionine transporter subunit N	methionine transporter	NP_414741.1	1 ± 0.076	15.86 ± 1.21
GlyA	serine hydroxy-methyltransferase	serine-glycine biosynthesis	NP_417046.1	1 ± 0.149	5.56 ± 0.829
SerA	phosphoglycerate dehydrogenase	serine-glycine biosynthesis	NP_417388.1	1 ± 0.025	25.5 ± 0.647
PepA	leucyl aminopeptidase	aminopeptidase	NP_418681.1	1 ± 0.295	5.42 ± 0.943

#### Enzymes involved in oxidative stress

In this study three enzymes involved in defence against oxidative stress, the manganese-containing superoxide dismutase SodA, the alkyl hydroperoxide reductase AhpC and the DNA starvation/stationary phase protection protein Dps were highly up-regulated with increasing temperatures from 37°C to 47.5°C. SodA showed a 17.7-fold, AhpC a 17.7-fold, and Dps a 7.3-fold up-regulation in the stress bioreactor at a cultivation temperature of 47.5°C compared to their level in the growth bioreactor.

**Figure 1:**
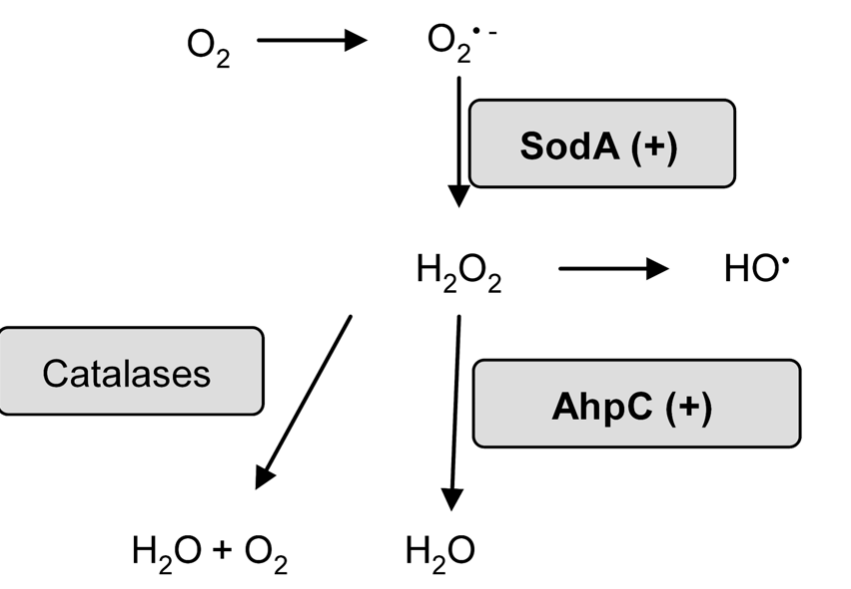
**Formation of reactive oxygen species and degradation**. The involved and identified proteins SodA and AhpC are up-regulated under higher cultivation temperatures in the stress bioreactor.

Under aerobic growth, reactive and toxic oxidative species are formed and can damage cell constituents like lipids, proteins and certain prosthetic groups of some enzymes and DNA [38-42]. Oxygen radicals such as O_2_^- ^and H_2_O_2 _evolve from the reduction of oxygen molecules. O_2_^- ^can be converted to H_2_O_2 _either spontaneously or by the superoxide dismutase (Sod). The formation of O_2_^-^, and consequently of H_2_O_2 _increased at elevated temperatures in the stress bioreactor and boosted the induction of SOD as an adaptive response to heat stress. H_2_O_2 _can be further decomposed by catalases or AhpC to yield H_2_O. Ahp, which is, is likely to be the primary scavenger of endogenous H_2_O_2_, i. e. the negative effect of the generated H_2_O_2 _can be averted by the scavenging activity of Ahp [43]. An overview of the oxidative species and their evolution is present in Figure 1.

Moreover, the Dps protein was 7.3-fold up-regulated in the cells cultivated in the stress bioreactor at 47.5°C. Dps protects genomic DNA against oxidative stress [44], nuclease cleavage, UV light and thermal stress, possibly by its DNA-binding ability to block the stress elements that attack DNA [45]. The genomic DNA is transformed by Dps among other proteins to a form of "nucleoid" to protect the genomic DNA against environmental stresses [46-49]. The results of our study indicate the existence of oxidative stress in the second bioreactor and the cell response to this stress is the simultaneous increase in the synthesis of AhpC and SodA in steady-state conditions.

#### Proteins of the cell envelope

The bacterial cell envelope serves as semi-permeable barrier between the cytoplasm of the microorganisms and the environmental medium performing a number of important functions. The cell membrane is involved in many growth and metabolic processes, e.g. location of the respiratory chain and the synthesis of the cell wall; it forms an osmotic gate, controls the entrance and the exit of substances and transmits the environmental signals. The cell wall is responsible for the cell shape and the outer membrane of *E. coli *constitutes the outermost area of the cell, which contains surface carbohydrate structures that are important virulence factors.

Five proteins of the cell envelope were found to be highly up or down-regulated. The L-fucosamine synthetase (FnlC) and the outer membrane protein A (OmpA) showed a down-regulation at high cultivation temperatures. In contrast, the outer membrane proteins F (OmpF) and C (OmpC), identified in one single spot, and the antigen 43 (Ag43) were up-regulated at a cultivation temperature of 47.5°C in the stress reactor under steady-state conditions. Ag43 is also located in the outer membrane of *E. coli*. The function of Ag43 has remained unknown until now. Previous studies indicated that the Ag43 protein mediates the autoaggregation of certain strains of *E. coli *in a liquid culture. Danese et al. reported that the Ag43 protein contributes to the *E. coli *biofilm formation in glucose-minimal medium, but not in complex broth [50]. Although no cell aggregation or biofilm formation as possible protective mechanisms were detectable in the stress bioreactor, we found that this protein is 7.6-fold up-regulated than under optimal growth conditions.

Contrastingly, the FnlC protein was down-regulated in the steady-state cultures at high temperature. This belongs next to FnlB, and FnlC to the *E. coli *O26 O-antigen gene cluster catalyzing a five-step reaction cascade in the biosynthetic pathway of the O antigen of the lipopolysaccharide layer (LPS) [51]. At 47.5°C no growth of *E. coli *could be detected and since less cell divisions occur lower amounts of cell constituents like the LPS, and consequently of the FnlC protein, are needed.

OmpA is associated with the peptidoglycan layer and has an important role in stabilizing the outer membrane and retaining the rod shape of the *E. coli *cell. The porin OmpA which was down-regulated at high temperatures is a major component of the outer membrane of *E. coli *[52]. Synthesis of OmpA is growth rate dependent [53], such that the ompA mRNA half-life increases proportionally with the growth rate [54]. The alternative sigma-factor σ^E ^fulfil a role in response to cell envelope stress and is essential for viability [14,55,56]. The majority of the genes which are under the control of σ^E ^are involved in synthesis, assembly and homeostasis of the outer membrane. The small noncoding RNA, MicA is positively regulated by the periplasmic sigma factor σ^E ^in response to envelope stress [57]. A transient expression of MicA leads to a strong reduction of the *ompA *mRNA level [58]. Envelope stress due to the accumulation of and aggregation of unfolded proteins thus reduces OmpA levels via σ^E ^and MicA under temperature stress. This periplasmic stress could cause an extremely reduced growth and be present in the cells during the non-growing maintenance phase.

The outer membrane proteins OmpC and OmpF [53,59,60] were found to be 30-fold up-regulated under steady-state heat shock conditions in the stress bioreactor at 47.5°C. Mecsas et al. identified overexpressed outer membrane porins (OMPs), such as OmpC and OmpF, as inducers of the σ^E ^activity[61]. Since the export of these OMPs to the periplasm was required for the σ^E ^induction, the overexpression of OMPs was assumed to generate a σ^E ^inducing signal shortly after passage through the inner membrane, perhaps as a consequence of the accumulation of unfolded periplasmic OMP species [61]. OmpF and OmpC play an important role in membrane transport: as they are water-filled, passive diffusion through channels allows hydrophilic molecules to cross the outer membrane [62]. In *E. coli*, the synthesis of the proteins OmpC and OmpF is controlled by the osmolarity as well as the temperature [53,62]. The wider diameter of the OmpF porin could be made responsible for an increased capacity of the cells for assimilation of nutrients by increasing the permeability of the outer membrane [63].

#### Chaperones and proteins involved in protein biosynthesis

Under temperature stress heat shock proteins are strongly induced to protect the cell against damage. Typical heat shock proteins are chaperones and proteases which operate in protein folding, refolding, quality control and protein degradation [11,12,64]. Eight proteins with chaperonic function or proteins involved in the protein biosynthesis showed different steady-state protein expression levels in the stress bioreactor. The chaperone protein Skp, the chaperone Hsp40, the leucyl-tRNA synthetase LeuRS and the ribosomal proteins S4, L5, and S11 were up-regulated at higher temperature culture conditions. Down-regulated protein expression levels showed the oligopeptide-binding protein OppA and the ribosomal protein S2.

The periplasmic chaperone Skp which has a general chaperone activity [65] and is under the control of the σ^E ^and the Cpx stress response [66]. σ^E ^is activated in response to unfolded OMPs due to the increased temperature [61,67], and consequently Skp is involved in the biogenesis and the folding of OMPs.

The oligopeptide-binding protein OppA interacts with unfolded and denatured proteins, such as the molecular chaperones. Richarme and Clada concluded that OppA is synthesized at similar rates before and several minutes immediately after heat shock, but only analysed the synthesis of OppA 16 minutes after heat shock[68]. In contrast, in our study OppA was found to be down-regulated at 47.5°C at the stress bioreactor under steady-state conditions, i.e. long lasting temperature stress at steady-state conditions seemed to cause a different stress response than a short term heat shock with the batch approach reported by Richarme and Caldas [68].

The third over-expressed protein Hsp40 (DnaJ) has been described as a molecular chaperone because of its ability to bind non-native polypeptides and prevent protein aggregation [69]. At high temperature of 47.5°C in the stress bioreactor several proteins may denature and unspecific chaperones like Hsp40 are therefore up-regulated to prevent their destruction under steady-state conditions.

Moreover, the leucyl-tRNA synthetase was found in higher concentrations under steady-state temperature stress than under optimal growth conditions. The tRNA synthetases catalyze the initial step of protein synthesis by covalently linking an amino acid to its cognate tRNA [70]. Under temperature steady-state stress conditions high amounts of heat shock proteins have to be synthesized, therefore requiring a higher synthesis of aminoacyl-tRNA synthetases.

Beside the synthetase, the expression profiles of several ribosomal proteins were also up-regulated under steady-state heat stress. In our study, we found the S4 and S11 protein of the ribosomal small subunit and the L5 protein of the large ribosomal subunit up-regulated in steady-state cultures at high temperatures. Ribosomal proteins are also known to have extra-ribosomal function [71] carried out through the interactions with RNA, DNA, or with other proteins. Kovacs *et al. *studied the assistance of RNA- and protein folding by ribosomal proteins [72]. They showed that the ribosomal proteins L15, L16, L18 and L19 not only presented RNA-chaperone activities, but that they are also potent protein chaperones with activities occasionally exceeding that of a classical protein chaperone, Hsp90. A possible chaperonic function of other ribosomal proteins has not been shown until now. Contrastingly only the S2 ribosomal protein of the small subunit was down-regulated at high temperatures in the steady-state cultures. The reason remains unclear.

#### Enzymes involved in the glycolysis, TCA cycle and mixed acid fermentation

**Figure 2:**
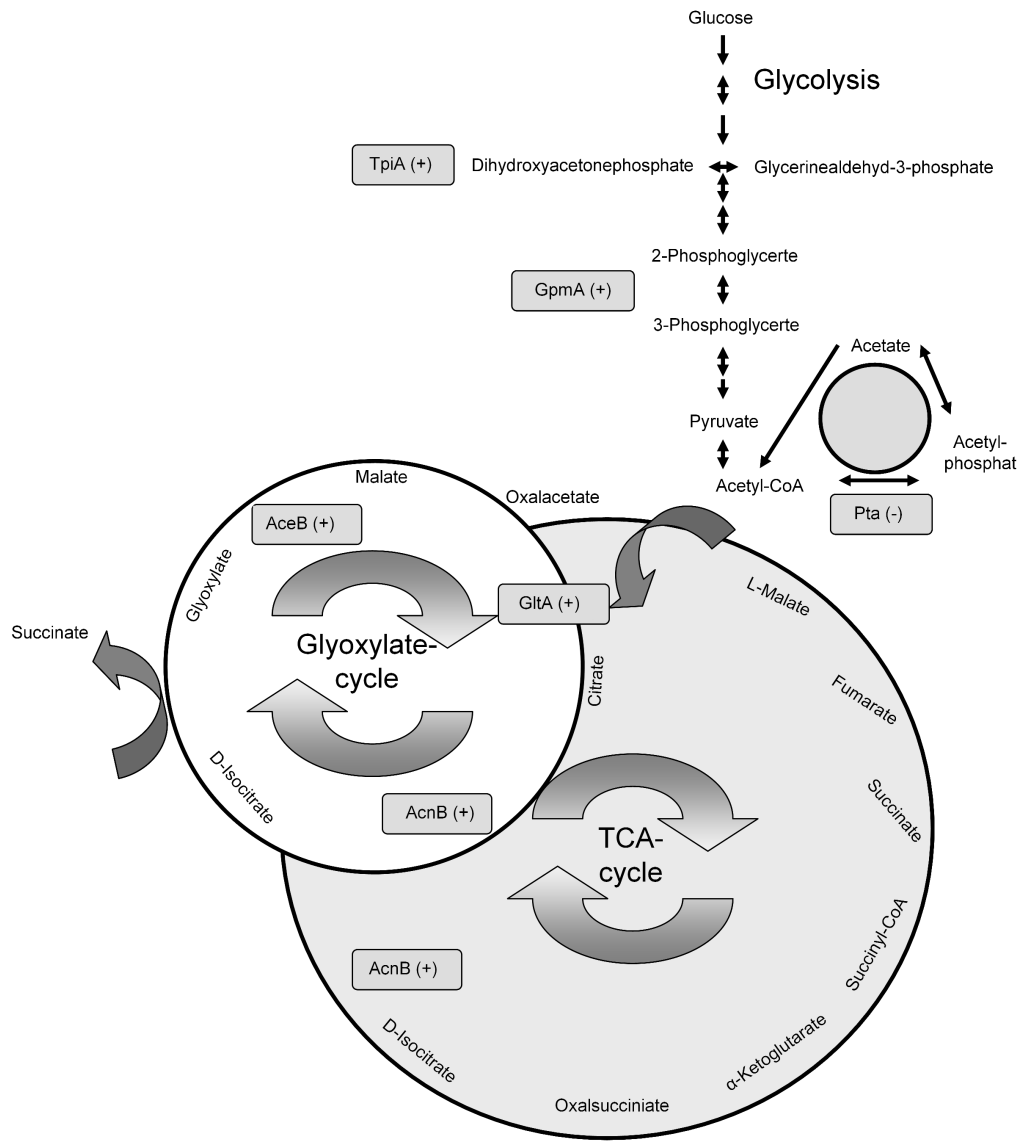
**Metabolic pathway of the gylcoylsis, the citrate cycle and the glyoxylate cycle**. The down or up-regulated enzymes are highlighted in gray.

Seven proteins involved in glycolysis, TCA cycle and mixed acid fermentation showed different expression levels at high temperatures in the stress bioreactor of our bioreactor cascade application. Two enzymes of the gylcolysis, the triosephosphate isomerase (TpiA) and the phosphoglycerate mutase (GpmA), one enzymes of the TCA cycle, the citrate synthase (GltA) and one enzyme of the glyoxylate cycle, the malate synthase (AceB) and the γ subunit of the ATP synthase were highly up-regulated at the cultivation temperature of 47.5°C The metabolic pathways where these different expressed proteins are involved are illustrated in Figure 2.

In the stress bioreactor at 47.5°C the glucose was completely consumed and high amounts of acetate were found. Acetate is synthesized by the glycolytic enzymes and the mixed acid fermentation occurs aerobically due to the growth of excess glucose [29,73]. This phenomenon is known as bacterial Crabtree effect [30,31,74,75]. In its undissociated or acidic form, this lipophilic weak acid easily permeates membranes, uncoupling the transmembrane pH gradient [35,36,76,77]. Once across the membrane, it dissociates into a proton and an anion [77,78]. The proton acidifies the cytoplasm, while the anion increases the internal osmotic pressure and interferes with methionine biosynthesis [79-81].

At 47.5°C really high amounts of acetate were measured. It seems that the non-growing cells were not able to assimilate the acetate due to the stationary phase inhibition of the expression of the acetylCoA-synthetase which is responsible for the uptake of acetate [82].

Aconitases catalyze the interconversion of citrate and isocitrate via cis-aconitate in the citric acid and glyoxylate shunt. Veit *et al. *showed a negative correlation of the *acnB *gene expression with acetate formation during continuous cultivation [83]. In the stationary phase the gene expression of the second aconitase AcnA increase and substitute the AcnB protein [84]. The *acnA *gene product shows a higher stability and affinity for citrate.

Besides the glycolytic pathway, another central metabolic pathway of aerobic organisms, the citric acid cycle, seems to be up-regulated at high temperatures in the stress bioreactor. The expression level of the citrate synthase indicates a higher demand of energy for the synthesis of heat shock proteins and intermediates in the cells cultivated under steady-state conditions in the stress bioreactor. The higher energy demand might also explain the 7-fold up-regulation of the γ subunit of the ATP synthase found in our study at a cultivation temperature of 47.5°C. The malate synthase encoded by the aceB gene belongs to the anaplerotic glyoxylate cycle and showed a 5.5-fold higher expression level in our study. The glyoxylate cycle, identified by Kornberg and Beevers [85], provides a simple and efficient strategy for converting acetyl-CoA into anaplerotic and gluconeogenic compounds. Studies of Kornberg, Krebs and Beevers identified two enzymes, isocitrate lyase and malate synthase, which, in conjunction with reactions of the citric acid cycle, allowed for the synthesis of anaplerotic succinate from two molecules of acetyl-CoA via a pathway named the glyoxylate shunt [85,86].

The acetyl-CoA acetyltransferase (Pta) could not be detected at a higher cultivation temperature of 47.5°C in the stress bioreactor. Temperature increases the Pta activity. Possibly, we found no higher expression of Pta because of the higher activity of Pta at higher temperatures; i. e. the regulation seemed not to have occurred on protein expression level. The results suggest the existence of a glucose starvation stress response in the cells of the second bioreactor which results in the observed up-regulation of TpiA and GpmA as well as an increase in the measured acetate production and excretion to the surrounding media. The balance between intracellular acetyl-CoA and extracellular acetate probably represents the most important influence on the intracellular acetyl-P pool. Pruess and Wolfe also demonstrated a correlation between incubation temperature and the intracellular acetyl-P pool [87]. At or below 34°C, they could not detect acetyl-P; above that temperature, the concentration increased. These results are consistent with the observation that extracellular acetate correlates with temperature [88] and can be readily explained by a reduced ackA transcription coupled with increased Pta activity [87], which also occurred in our experimental setup.

#### Enzymes involved in amino acid biosynthesis

Amino acids play a very important role as building block of proteins. Especially at high temperatures many new proteins have to be synthesized or degraded to adapt and survive the stress conditions. Under heat stress several enzymes of the amino acid biosynthesis were found to be up-regulated in steady-state cultures. The 5-methyltetradyropteroyltriglutamate-homoserine methyltransferase (MetE), the thiosulfate transporter (CysP), the DL-methionine transporter subunit (MetN), the DL-methionine transporter subunit (GlyA), the phosphoglycerate dehydrogenase (SerA) and the leucyl aminopeptidase (PepA) were highly up-regulated with increasing temperatures. The GTP cyclohydrolase I (FolE) is the only protein involved in amino acid biosynthesis which was down-regulated.

At high cultivation temperature many proteins denature and are degraded by proteases. Under steady-state heat shock conditions we found the aminopeptidase PepA 5.4-fold up-regulated. The PepA enzyme displays strong thermostability and is active mainly on large peptides and is thus capable of degrading proteins (reviewed in [89]). In the present case, most probably the regenerated amino acids coming from degraded proteins served as building blocks for the synthesis of new proteins.

In *E. coli*, the growth rate at elevated temperatures is controlled by the availability of endogenous methionine, which is limited because of the temperature sensitivity of the *metA *gene product, the homoserine transsuccinylase, the first enzyme of the methionine biosynthesis [79-81].

To balance the methionine limitation the cells can take, for example, the methionine released from the already died cells. In any case under steady-state conditions the D/L-methionine transporter subunit (MetN) was 15.9-fold up-regulated at 47.5°C most probably to facilitate methionine uptake. MetN is the putative ATPase and MetI is the membrane-spanning region of the MetD ABC transporter. Kadner (1974, 1975) had shown that growth conditions can cause several-fold changes in the amount and activity of the methionine transport system of *E. coli *[90,91]. Cells grown without methionine exhibited a higher initial rate of uptake than those grown with additional methionine [90].

Besides the assimilation of methionine the alternative methionine synthesis pathway via serine seems to the up-regulated as well. That is the case of the MetE protein which catalyses the final methyl transfer to homocysteine to form methionine. This showed a 9.7-fold higher expression level at 47.5°C than in the growth bioreactor at 37°C. The MetE enzyme has a low catalytic turnover and accounts a large percent of the total soluble protein in cells grown on minimal medium (reviewed in [92]). However, at a high cultivation temperature in the stress bioreactor the synthesis of methionine seems to be limited although the *metE *gene expression is not repressed.

Additionally, two enzymes of the serine/glycine synthesis pathway were highly up-regulated at 47.5°C in the stress bioreactor. The 3-Phosphoglycerate dehydrogenase (*serA *gene product) showed a 25.5-fold higher expression level at 47.5°C in cells of the stress bioreactor compared to those cultivated in the growth bioreactor. Moreover the *glyA *gene that encodes for the serine hydroxymethyltransferase was 5.6-fold up-regulated as well. These two enzymes are involved in the serine-glycine pathway which is important for the availability of serine and glycine to synthesize proteins under heat shock conditions. Serine is used in the synthesis of cysteine, tryptophan, and phospholipids and can also be converted to glycine and a C_1 _unit by serine hydroxymethyltransferase [93,94]. Glycine is a precursor of purines and heme-containing compounds [95-97]. C_1 _units are used in the synthesis of purines, histidine, thymine, methionine, the formylation of the aminoacylated initiator tRNA, and S-adenosylmethionine (SAM) [95]. SerA and GlyA are both also involved in methionine biosynthesis to balance the endogenous methionine limitation.

**Figure 3:**
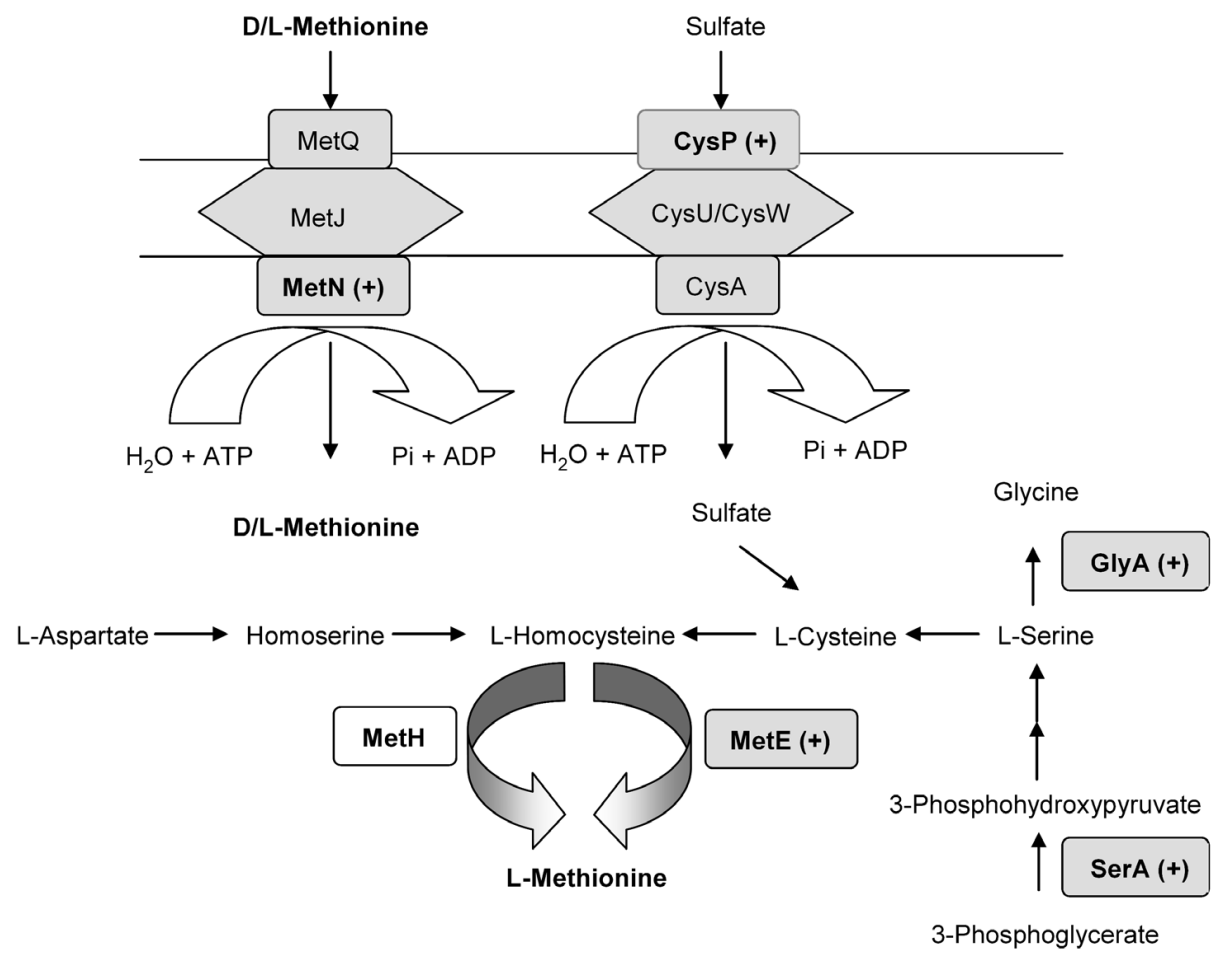
**Metabolic pathway of the amino acids methionine, serine, cysteine and glycine and the uptake of extracellular methionine and sulfate**.

The CysP protein is part of the periplasmic transport system for the uptake of sulphate and thiosulfate released from cells undergoing lysis. At 47.5°C the CysP protein showed a 4.2-fold up-regulation. Cysteine and methionine are essential building blocks of proteins and must be along with the sulfur-containing cofactors thiamine, lipoic acid, biotin, molybdopterin, glutathione, coenzyme A and coenzyme M, synthesized by the cell or recruited from the environment as inorganic or organic form [98]. The uptake systems and pathways where these different expressed proteins are involved are illustrated in Figure 3.

In contrast the FolE protein, the GTP-Cyclohydrolase I, showed a heat-induced decrease. At 37°C in the growth bioreactor 20.3-fold higher amounts of FolE could be detected with respect to the stress bioreactor at 47.5°C. FolE is the first enzyme of the de novo tetrahydrofolate biosynthetic pathway in *E. coli*. Folic acid, in the form of various tetrahydrofolate (THF) derivatives, serve as cofactor in one-carbon transfer reactions during the synthesis of purines, thymidylate, pantothenate, glycine, serine, and methionine [99]. Lee *et al. *found the highest specific activity of the GTP-Cyclohydrolase I of *E. coli *at 60°C [100]. In our study the GTP-Cyclohydrolase I is probably up-regulated at lower temperatures in the growth bioreactor and down-regulated i.e. most probably partially degraded in the stress bioreactor because of its higher specific activity with increasing cultivation temperatures.

## Conclusion

In the present work a cascade of two continuously operated bioreactors was used to study the steady-state heat shock response of *E. coli *to temperature up-shift. The temperature stress response was investigated by means of 2-D gel electrophoresis. The bioreactor system configuration allowed the analysis of the stress response under well-defined conditions in steady-state cultures, which delivered a different protein spectrum compared to standard batch cultures in shaking flasks and bioreactors. Setting a high cut-out spot-to-spot size ratio of 5, proteins involved in defence against oxidative stress, cell envelope proteins, chaperones and proteins involved in protein biosynthesis, the energy metabolism and the amino acid biosynthesis were found to be differently expressed at the high cultivation temperature.

Due to the higher cultivation temperature the synthesis of oxygen radicals increased and the cells protected their cellular components up-regulating SodA, AhpC and Dps at a cultivation temperature of 47.5°C in the stress bioreactor. Especially the existence of oxidative stress may have a great influence in the new design or further optimization of heterologous protein production processes using temperature-induced systems. Concretely, after induction of the protein expression through a temperature shift, both the agitation and the aeration rate should be reduced to minimize the oxidative stress; this action might have the additional advantage of a possible redirection of the protein synthesis resources to the desired product. Contrastingly to batch experiments Privalle et al. only showed that induction of SodA in *E. coli *was caused by the exposure to 48°C in shaking flask [101]. Moreover, Benov and Fridovich [102] investigated the oxidative stress under heat shock in batch experiments too and showed the essentiality of SodA: the exposure of a superoxide dismutase-null strain of *E. coli *to 45°C to 48°C led to a profound loss of viability [102].

The higher expression of outer membrane proteins and the up-regulation of the glycolysis, the TCA cycle and the γ subunit of the ATP synthase indicated a higher energy demand at a cultivation temperature of 47.5°C compared to the reference cultivation at 37°C. In contrast to our results, in batch cultivations, non-growing cells enter the stationary phase followed by a down-regulation of enzymes of the central metabolic pathways.

At high temperatures the growth rate is markedly influenced by the availability of exogenous methionine [18]. In the absence of methionine, growth stops at 45°C. To compensate the methionine requirement in steady-state cultures the PepA extracelullar aminopeptidase, the GlyA and the SerA proteins which are part of the serine-g lycine pathway, as well as the methionine transporter subunit MetN and the methionine synthetase MetE were up-regulated at high cultivation temperatures in the stress bioreactor. CysP is also up-regulated and is responsible for the uptake of sulphate and thiosulfate which are needed for the synthesis of the essential building blocks methionine and cysteine. These results agree with batch experiments reported elsewhere. An amino acid limitation via the ppGpp mediated stringent response caused an up-regulation of the amino acid biosynthesis and a down-regulation of transcription and translation [103]. In our study, the amino acid biosynthesis seems to be up-regulated too. However, in the continuously operated bioreactor cascade we found an up-regulation of ribosomal proteins and the leucyl-tRNA-synthetase.

In our experiments glucose was present in limiting concentrations causing an up-regulation of proteins for the uptake of alternative C-sources. At 47.5°C nearly no growth could be detected although almost all glucose was consumed and high amounts of acetate were measured. The existence of starvation stress proteins may be better interpreted as an *E. coli *glucose insufficiency signal in our system. The assimilated glucose was most probably used for maintenance metabolism to adapt the stress conditions and to synthesize all the proteins and intermediates needed for protection and survival. Particularly considering the excess of an alternative C-source, acetate, the high energy demand can be most probably attributed to the synthesis of heat shock proteins, ribosomal proteins and aminoacyl-tRNA synthetases. Under these conditions the gyloxylate cycle was up-regulated at high cultivation temperatures. At 47.5°C the non-growing cells were in an stationary phase like stadium and were not able to assimilate the acetate due to an inhibition of the expression of the acetylCoA-synthetase which is responsible for the uptake of acetate [82].

The analysis of the stationary *E. coli *heat shock response showed the existence of different stress factors in a continuously operated system. Identical to batch experiments we found a protease, several chaperones, proteins of the amino acid biosynthesis and the oxidative stress response highly up-regulated in the stress bioreactor at 47.5°C. Interestingly, proteins of the central metabolic pathways and the protein biosynthesis were up-regulated in the continuously bioreactor system compared to batch experiments reported elsewhere. Since the heat shock response is not only a function of the stress intensity but also a function of the duration of stress, our two bioreactors platform offers a remarkable possibility to investigate stress intensity and duration, independently from each other, varying temperature level and dilution rate (mean residence time) respectively.

## Methods

### Bacterial strain and media

The *Escherichia coli *strain MG1655, a wild-type K12 strain, was used in this study. For the cultivations the defined minimal media M1, containing the following components, were utilized: 1.25 mM NH_4_Cl, 5.05 mM (NH_4_)_2_SO_4_, 7.96 mM KH_2_PO_4_, 4.78 mM K_2_HPO_4_, 30.6 mM NaH_2_PO_4_, 55.5 mM C_6_H_12_O_6_•H_2_O, 0.068 mM CaCl•2H_2_O, 0.00174 mM ZnSO_4_•7H_2_O, 0.00148 mM CuCl_2_, 0.0148 mM MnSO_4_•H2O, 0.0074 mM CoCl_2_, 0.002 mM H_3_BO_3_, 0.0104 mM AlCl_3_•6H_2_O, 0.002 mM Na_2_MoO_4_•2H_2_O, 0.036 mM FeSO_4_•7H_2_O, 4.16 mM MgSO_4_•7H_2_O.

### Growth conditions

**Figure 4:**
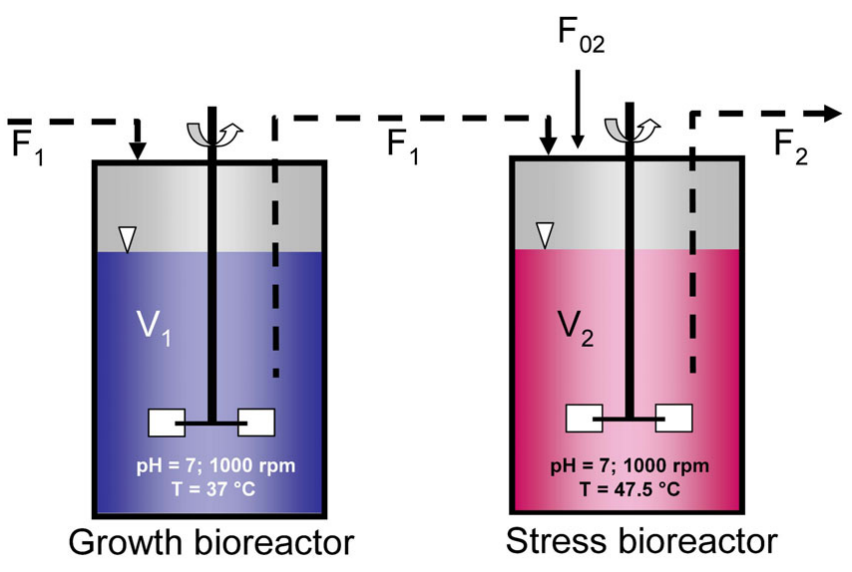
**Experimental set-up with a cascade of two continuous stirred tank reactors connected in series**. The dilution rate D_1 _of the growth reactor was kept constant at 0.225 h^-1 ^adjusting the fresh media stream F_0_. The dilution rate D_2 _of the stress bioreactor was also kept constant, but it amounted 1.1 * D_1 _due to the use of a second fresh substrate feed F_02_, together with the reactor stream F_1_.

*E. coli *was cultivated in a cascade of two continuous stirred tank reactors connected in series (Figure 4). Two 3 L-Applikon bioreactors (Applikon, AC Schiedam, The Netherlands) with a working volume of 2 L were used. The Applikon-bioreactors were run and controlled by an ADI 1010 Bio Controller, an ADI 1025 Bio Console and an ADI 1032 Stirrer Controller (Applikon). The cultivation parameters pH, dissolved oxygen, temperature and agitation were monitored using the software BioXpert, v.2.70.115 (Applikon). The exhaust gas analysis was performed with the BCP-O_2 _and BCP-CO_2 _gas sensors (BlueSens, Herten, Germany) and the associated software FermVis (BlueSens).

Prior to the start of a continuous cultivation the growth-bioreactor was inoculated directly from a glycerol stock of *E. coli *MG1655 and cultivated in batch at 37°C, 1000 rpm, pH 7.0 and aeration of 3 L min^-1 ^air. Biomass concentrations were estimated measuring the optical density of the culture broth at 660 nm (OD_660_) with the UV-Vis spectrophotometer Helios α (Thermo Fisher Scientific Inc. Waltham MA, USA). After reaching an OD_660 _of approximately 0.5 the continuous cultivation was started. Figure 8 shows the experimental set-up of the bioreactor cascade. In the cascade of continuously operated bioreactors, the dilution rate *D*_1 _of the growth reactor was kept constant at 0.225 h^-1 ^adjusting the fresh media stream *F*_0_. The dilution rate *D*_2 _of the stress reactor was also kept constant, but it amounted 1.1 *D*_1 _due to the use of a second fresh substrate feed *F*_02_, together with the reactor stream *F*_1_. The second feed *F*_02 _containing 10-fold concentrated media served as nutrients source for the cells growing in the second bioreactor to avoid any substrate limitation. In the first reactor the cultivation temperature was set to 37°C, while in the second bioreactor, acting as stress unit, the temperature was set to a higher temperature of 47.5°C. The pH was controlled in both reactors using 6.25% NH_3 _and 9.25% HCL. Dissolved oxygen concentration was kept above 20% and antifoam agent was added every 6 h for 1 min using a timer. Biomass was monitored using the measured OD_660 _of the samples taken at regular time intervals. Concentration of glucose was quantified using the glucose analyzer 2300 STAT Plus (Ysi, Yellow Springs, Ohio, USA). Secreted metabolites were determined using HPLC analysis. A D-7000 system by Merck-Hitachi was used with a RI-detecor (L-7490). For detection of small carboxylic acids an ion-exchange-column Polysphere OA HY (Merck, Darmstadt, Germany) was chosen with 5 mM H_2_SO_4 _as the mobile phase. The establishment of steady-states in both reactors was monitored via exhaust gas analysis. Constant oxygen uptake and carbon dioxide production rates after five residence times in the respective reactor confirmed the steady-state conditions.

For 2-D gel analysis cell samples were taken during steady-state conditions and were immediately cooled down at 4°C in five times the sampling volume of phosphate-buffered saline (PBS) solution. To stop the protein biosynthesis 1 mg mL^-1 ^Chloramphenicol was added. Finally the cells were separated by centrifugation at 6.500 g for 15 min at 4°C, washed twice with phosphate-buffered saline (PBS) solution and stored at -20°C until further use.

### Extraction and separation of *Escherichia coli *proteins by two-dimensional gel electrophoresis

To obtain raw protein extracts cell pellets were resuspended in a lysis buffer containing 7 M urea, 2 M thiourea, 4% (w/v) CHAPS, 1% (w/v) dithiothreitol (DTT), 0.5% (w/v) amidosulfobetaine-14 (ASB-14), 0.5% (v/v) Triton X-100, 0.8% (w/v) Pharmalyte™ pH 3-10, and 5 mM Pefabloc. The cells were disrupted by ultrasonication in an ice bath for 2 min with the Sonifier S-250 D (Branson Ultrasonics Corp., Danbury, Connecticut, USA) applying an interval pause each 0.5 s with amplitude of 30%. Insoluble cell components were separated by centrifugation at 13.000 g for 15 min at 4°C. To remove DNA and RNA components a phenol precipitation and subsequent acetone extraction resulted in the best 2D-gel electrophoresis. The proteins were extracted with TE-buffer (10 mM EDTA, pH 7.4) saturated phenol. Proteins formed a white interphase between the phenolic and the aqueous phases. Subsequently the precipitated proteins were separated by centrifugation at 13.000 g for 15 min at 4°C, the protein pellets were washed twice with cold acetone (-20°C), dried under vacuum (speed vac) and stored at -20°C until use. The extracted proteins were dissolved in rehydration buffer (7 M urea, 2 M thiourea, 4% (w/v) CHAPS, 1% (w/v) DTT, 0.5% (w/v) ASB-14, 0.5% (v/v) Triton X-100, 0.5% IPG buffer pH 3-10 and trace amount of bromphenol blue). Insoluble components were separated by centrifugation at 13.000 g for 15 min at 4°C. The protein concentration measurement was carried out using the 2D-Quant-Kit (Amersham Bioscience, Buckinghamshire, GB) according to the manufacturer's instructions. 250 μg protein of each sample were applied to the first-dimensional gel electrophoresis of isoelectric focusing (IEF) using Immobiline DryStrip gels of pH 3-10 (Bio-Rad, Hercules, USA) by in-gel rehydration in a PROTEAN IEF Cell Isoelectric Focusing System (BioRad, Hercules, California, USA) at 20°C and with the following settings: Rehydration: 50 V, 12 h; 200 V, 1 h; 500 V, 1 h; 1000 V, 1 h; linear gradient from 1000 V to 10000 V within 0.5 h; 10000 V, 8 h. For each sample duplicate gels were analysed. Subsequently the IPG strips were consecutively equilibrated in the SDS Equilibration buffer 1 (6 M urea, 0.05 M Tris-HCl pH 8.8, 2% (w/v) SDS, 30% glycerol, 1% (w/v) DTT) and SDS Equilibration buffer 2 (6 M urea, 0.05 M Tris-HCl pH 8.8, 2% (w/v) SDS, 30% Glycerol, 2.5% (w/v) iodacetamide and trace amount of bromphenol blue) for 15 min. The SDS-PAGE, the second dimensional gel electrophoresis, was carried out using a 12.5% gel (12.5% acrylamide T-30%, 0.375 M Tris-HCl pH 8.8, 10% (w/v) SDS, 10% (w/v) ammonium persulfate (APS), 10% (v/v) tetramethylethyleneamine (TEMED)) and separated at 25°C with the following settings: 2 W/gel for 1 h, 100 V 20 h until the bromphenol blue dye front reached the bottom of the gel. The proteins were locked into position by the use of a fixation-solution (30% (v/v) Ethanol, 10% (v/v) acetic acid) overnight. Afterwards the gels were stained with the fluorescent dye ruthenium-II-bathophenanthrolin disulfonate (RuPBS) [104] for 6 h and scanned with PharosFX (Bio-Rad). The analysis of the protein spot intensity, which is defined as the ratio of the single spot volume to the total spot volumes of all protein spots on a 2D-gel, and the characterization of the expression changes of proteins was performed with the DELTA 2D Sofware, Version 3.4 (Decodon, Greifswald, Germany).

#### Enrichment of membrane proteins using triton-X-114

The protein samples were prepared according to the method described by Bordier [105]. Briefly, after cell disruption by ultrasonication, the samples were centrifuged in a Beckman 70.1 Ti rotor (Beckman-Coulter, Fullerton, California, USA) at 170.000 g for 1 h at 4°C. The proteins of the separated membrane components were extracted using 10 mM Tris-HCl, pH 7.4, 150 mM NaCl, and 0.5 and 1.0% Triton-X-114 at 0°C. The samples were overlaid on a cushion of 6% (w/v) sucrose, 10 mM Tris-HCl, pH 7.4, 150 mM NaCl, and 0.06% Triton-X-114, incubated 3 min at 30°C and centrifuged at 300 g for 3 min at room temperature. The receiving oily droplet was retained and the supernatant was extracted using 0.5% Triton-X-114 as described above. The second droplet coming from the extraction using 1.0% Triton-X-114 was unified with the first droplet and stored at -20°C until use for the 2D-gel analysis.

#### Enrichment of membrane proteins using sodium carbonate treatment

After cell disruption by ultrasonication, the samples were centrifuged in the Beckman 70.1 Ti rotor (Beckman-Coulter) at 170.000 g for 1 h at 4°C. The membrane proteins were enriched according to the method described previously in Fujiki *et al*. [106]. The membrane pellet was diluted in 6 ml of 100 mM sodium carbonate, pH 11.5 and incubated at 0°C for 30 min. The samples were centrifuged in polycarbonate tubes at 4°C for 1 h at 170.000 g. The dissolved proteins in the supernatant were precipitated using 10% TCA, washed twice using ice cooled acetone and stored at -20°C until use for the two-dimensional gel electrophoresis. The receiving membrane pellet after ultracentrifugation was extracted again using 100 mM sodium carbonate, incubated at 0°C for 30 min and centrifuged at 4°C for 1 h at 170.000 g. The proteins in the supernatant were precipitated again using 10% TCA.

#### Enrichment of membrane proteins with the aid of a rehydration buffer

The cells were dissolved in lysis buffer and disrupted by ultrasonication. Afterwards, the samples were centrifuged in the Beckman 70.1 Ti rotor (Beckman-Coulter) at 170.000 g for 1 h at 4°C. The receiving membrane pellet was resuspended in rehydration buffer (7 M urea, 2 M thiourea, 4%(w/v) CHAPS, 1% (w/v) DTT, 0.5% (w/v) ASB-14, 0.5% (v/v) Triton X-100, 0.5% IPG buffer pH 3-10 and trace amount of bromphenol blue) and used for two-dimensional gel electrophoresis.

### Protein analysis by mass spectrometric analysis and identification using protein databases

Protein spots showing more than 5-fold increase or decrease in spot intensity were cut off from the 2D-gels and digested with trypsin according to a method described previously [107]. The digested proteins were extracted and purified with reversed-phased C18 ZipTips (Millipore, Bedford, Massachusetts, USA). Matrix-assisted laser desorption ionization time-of-flight mass spectrometry (MALDI-TOF MS) with a Bruker Ultraflex time-of-flight mass spectrometer (Bruker Daltonik GmbH, Bremen, Germany) was carried out as described by Wang *et al. *[107]. To identify the proteins the public protein sequence database Mascot (Matrix Science Ltd., UK, ) was consulted using the public databases NCBInr and SWISS-PROT/TrEMBL. The following settings were used for the identification: two missed cleavage sites were allowed, cysteine was carbamidomethylated and methionine was allowed to be partially oxidized.

## Competing interests

The authors declare that they have no competing interests.

## Authors' contributions

SL performed 2-DE experiments, sample digestion, preparations and protein enrichment, image analysis, protein identification and participated in the writing of the manuscript. CF carried out the bioreactor experiments, extracellular metabolite measurements and revised the final manuscript. EFL participated in experimental design, data analysis, coordination and preparation of the final version of the manuscript. All authors read and approved the final manuscript.
